# Parents’ employment and non-chromosomal congenital anomalies in Korea: a national population cohort study

**DOI:** 10.4178/epih.e2025018

**Published:** 2025-04-10

**Authors:** Kyuwon Kim, Hoyol Jhang, Erdenetuya Bolormaa, Chae Bong Kim, Seung-Ah Choe

**Affiliations:** 1 Department of Public Health, Korea University School of Public Health, Seoul, Korea; 2 Department of Health and Medical Administration, University of Jae-Neung, Incheon, Korea; 3 Department of Preventive Medicine, Korea University College of Medicine, Seoul, Korea; 4 Research and Management Center for Health Risk of Particulate Matter, Korea University, Seoul, Korea

**Keywords:** Pregnancy, Occupation, Congenital malformation

## Abstract

**OBJECTIVES:**

We assessed the association between parents’ employment status, including industrial classification, and non-chromosomal congenital anomalies in offspring.

**METHODS:**

We analyzed data from mothers who delivered live births between 2020 and 2022, linking their records with those of their neonates from the National Health Information Service (NHIS) database. Our analysis focused on common industrial classifications representing at least 6% of the total workforce. Congenital anomalies were identified based on neonates’ diagnostic codes. We conducted logistic regression to estimate odds ratios (ORs) of congenital anomalies by the industrial classification of mothers and their partners, adjusting for individual risk factors, with the financial industry serving as the reference category.

**RESULTS:**

Among 338,637 women with a live birth, 148,818 (43.9%) were employed at the time of pregnancy. Employment was associated with a higher risk of congenital anomalies (OR, 1.08; 95% confidence interval [CI], 1.04 to 1.12). Within the common industrial classifications, health and social work exhibited the highest risk (OR, 1.11; 95% CI, 1.06 to 1.22) compared to the financial industry. Women employed in general hospitals showed particularly elevated risks (OR, 1.19; 95% CI, 1.04 to 1.37). Among male partners, the risk estimates were generally imprecise.

**CONCLUSIONS:**

The study indicates that certain industries are linked with a higher risk of congenital anomalies among women workers. These findings underscore the need for enhanced safety measures in high-risk industrial settings to reduce the occurrence of congenital anomalies.

## GRAPHICAL ABSTRACT

[Fig f1-epih-47-e2025018]

## Key Message

• Among 338,637 women who delivered between 2020 and 2022, 43.9% were employed during pregnancy, and employment status was associated with an increased risk of non-chromosomal congenital anomalies (OR 1.08; 95% confidence interval [CI] 1.04-1.12).

• Compared to the financial industry, health and social work showed the highest risk (OR 1.11; 95% CI 1.06-1.22), with women working in general hospitals exhibiting an even greater elevated risk (OR 1.19; 95% CI 1.04-1.37).

• Risk estimates by industrial classification for male partners were generally imprecise, with wide confidence intervals.

## INTRODUCTION

Congenital anomalies occur in approximately 2-3% of live births globally [1], with variations across regions and populations. These anomalies remain a significant public health concern because they can lead to long-term disability and adversely affect the quality of life for affected individuals and their families. The factors contributing to congenital anomalies are multifaceted, including genetic, environmental, and maternal health factors [2]. Among these, industrial exposure to hazardous substances during pregnancy has attracted growing attention for its potential to disrupt fetal development and lead to adverse outcomes such as miscarriages, stillbirths, and congenital anomalies [3].

Male and female workers of reproductive age often encounter various chemical, biological, and physical hazards in the workplace that can increase the risk of adverse pregnancy outcomes. These hazards include substances such as lead, mercury, and other heavy metals, as well as biological agents like viruses [4]. Physical hazards, including radiation and ergonomic stress, may also contribute to reproductive health issues, leading to complications such as infertility, spontaneous abortion, preterm labor, and low birth weight. Despite the increasing participation of women in the workforce, many remain concerned about the potential health risks associated with their industrial environments, particularly regarding reproductive health and fetal development.

The traditional focus on maternal exposures might overlook potential paternal contributions. Sperm are susceptible to damage from environmental toxins and heat. Moreover, because most male adults of reproductive age are employed, paternal occupational exposures can be diverse and potentially harmful. Previous studies have examined a limited range of industries or occupational exposures expected to have a higher risk for adverse birth outcomes. We assessed the association between the industrial classifications of pregnant women and their male partners using a national population cohort. This approach captures a broader spectrum of potential hazards encountered by employed women and their male partners. The primary objective of our study was to identify high-risk industries for congenital anomalies in offspring, thereby providing evidence to inform policies aimed at mitigating transgenerational health risks.

## MATERIALS AND METHODS

### Data source and study cohort

We conducted a population-based cohort study in Korea by analyzing data on pregnant women and their newborns from the National Health Information Service (NHIS) database [5]. Our customized dataset contains identification numbers that link women who had a live birth with their newborns from 2020 to 2022. The NHIS covers 50 million people, approximately 97% of the Korean population, and includes individual-level data on age, gender, residential area, disability status, income level (as reflected by the NHIS subscription premium), employment status, industrial classification of employment, and all healthcare utilization records. Our eligibility criteria included women with a treatment code for live birth who were between 20 years and 49 years old at the time of delivery. Medical beneficiaries and self-employed individuals were excluded. A total of 339,824 women with employee NHIS subscriptions (either as employees or as dependents of employed individuals) were identified as having a live birth during 2020-2022. After excluding women aged <20 years or >49 years, the final study population comprised 338,637 women (Supplementary Material 1).

### Exposure variables

The study population was initially divided based on employment status. Since the NHIS data provides industrial classification only for workplace subscribers, information on the employed industry of women’s male partners is available solely for women who are not employed. The association between the industrial classification of the male partner and congenital anomalies was analyzed among women who were not employed during pregnancy. The NHIS database utilizes an industrial classification system similar to the International Standard Industrial Classification of All Economic Activities revision 4.0 [6]. The individual categories of the Korean Standard Industrial Classification have been aggregated into 17 sections: (1) agriculture and forestry; (2) fishing; (3) mining and quarrying; (4) manufacturing; (5) electricity, gas, steam, air conditioning supply, water supply, sewerage, waste management, and remediation; (6) construction; (7) wholesale and retail trade and repair of motor vehicles and motorcycles; (8) accommodation and food service activities; (9) transportation and storage; (10) financial and insurance activities; (11) real efstate activities; (12) public administration, defense, and compulsory social security; (13) education; (14) human health and social work activities; (15) administrative and support service activities; (16) activities of households; and (17) activities of extraterritorial organizations and bodies. These 17 sections are further categorized into 711 divisions. For example, the education section includes industries such as education service, primary education institutions, secondary education institutions, higher education institutions, unclassified education institutions, early childhood education institutions, primary schools, general secondary education institutions, technical and vocational secondary education institutions, higher education institutions, special schools, foreign schools, office-related education institutions, technical and vocational training academies, general tutoring academies, and other educational institutions.

As of 2020, 97.7% of all births in Korea occurred within legal marriages, and all marital couples registered in the NHIS during the study period were in different-sex marriages [7]. Therefore, the industrial classification for women who are dependents of employed subscribers can be considered reflective of their male partners’ employment industries. To identify high-risk industries for congenital anomalies, our analysis was restricted to common industrial sections representing at least 6% of employed women and their male partners—equating to over 6,000 employees in both groups. For the industrial divisions, those with over 1,500 employees were selected among employed women and their male partners.

### Outcome variables

The outcome variables in this study were based on medical records of children diagnosed with congenital anomalies after birth. Given the wide range of possible congenital anomalies, the analysis focused on major congenital anomalies to target conditions with significant potential impacts on a child’s health and development [8]. The list of major congenital anomalies was based on diagnostic codes defined by the updated guideline of the European Surveillance of Congenital Anomalies (EUROlinkCAT) [9]. Minor malformations that do not result in critical functional or clinical outcomes (such as saddle nose [Q189], congenital pes planus [Q665], and dolichocephaly [Q672]) were excluded. Chromosomal anomalies (Q90-Q92, Q93, Q96-Q99) were excluded to concentrate on environmental factors in the workplace that may influence fetal development. Genetic syndromes were also excluded because they are likely inherited or result from abnormalities in germ cells or fertilized eggs prior to conception, making it difficult to directly associate them with occupational exposure during pregnancy.

### Covariates

Our explanatory model included the mother’s age at childbirth, residence in the Seoul capital area, smoking history, pre-pregnancy obesity, relative income level, presence of disability, and obstetric comorbidities. The age of pregnant women was categorized into five-year intervals (20-24, 25-29, 30-34, 35-39, ≥40). For income, percentiles were grouped into four categories for analytical convenience: most deprived (0-30%), moderately deprived (30-60%), less deprived (60-80%), and least deprived (richest: 80-100%). Residence in the Seoul capital area—which includes Seoul, Incheon, and Gyeonggi Province—was used as a proxy for high socioeconomic status, as the area is a central urban region with high living costs and readily accessible healthcare services. Pre-pregnancy obesity was defined as a body mass index >25 kg/m². Because the data did not include clinical information on male partners, only maternal covariates were used in the model.

### Statistical analysis

The analysis began with descriptive statistics to compare the general characteristics of women who were employed versus those who were not during pregnancy. We calculated crude and adjusted odds ratios (ORs) for non-chromosomal congenital anomalies by employment status and industrial category for women, consistently using the financial industry as the reference group. Risk estimates were adjusted for maternal age, pre-pregnancy obesity, smoking history, residential area, relative household income, presence of maternal disability, and obstetric comorbidities, including gestational diabetes and hypertension. Comparative analyses for high-risk industrial sections and divisions were conducted only among employed women because all male partners of non-employed women were employees in our study population. The industrial section and division with the lowest risk of congenital anomalies served as the reference group. All statistical analyses were performed using SAS version 9.4 (SAS Institute Inc., Cary, NC, USA).

### Ethics statement

This study was exempt from review by the Korea University Institutional Review Board (exemption No. KUIRB-2022-0165-01).

## RESULTS

Among the 338,637 women who gave birth, 148,818 (43.9%) were employed at the time of pregnancy (Table 1). Women who were employed during pregnancy were more likely to be younger (median age 32 vs. 33 years), reside in the Seoul capital area (55.4 vs. 50.0%), have a history of smoking (2.1 vs. 1.7%), and be pre-pregnancy obese (11.8 vs. 6.2%). They were also more likely to have a relatively lower income (28.4% in the lowest quartile vs. 19.1% among non-employed women). In contrast, disability (less than 0.1 vs. 0.5%) and obstetric comorbidities (38.5 vs. 44.3%) were more common among women who were not employed during pregnancy.

Overall, 23,036 cases (6.8%) of non-chromosomal congenital anomalies were identified. Employment during pregnancy was associated with a higher risk of non-chromosomal congenital anomalies (adjusted OR, 1.08; 95% confidence interval [CI], 1.04 to 1.12; Table 2). Among employed women, seven industrial sections were prevalent: manufacturing; wholesale/retail trade; financial and insurance activities; real estate, renting, and business activities; education; health and social work; and public/social/personal services. In these common industrial sections, health and social work demonstrated the highest risk for congenital anomalies, with an OR of 1.19 (95% CI, 1.04 to 1.37) compared to financial and insurance activities. The comparative risk estimates for congenital anomalies in the other common industrial sections were generally imprecise.

Within the industrial divisions, the most common among employed women were financial intermediation, general hospitals, pre-primary education, general public service, manufacture of office machinery and equipment, primary schools, and child daycare services. When compared to the industrial division of financial intermediation, which had the lowest risk of congenital anomalies (4.7%), women employed in general hospitals (OR, 1.19; 95% CI, 1.04 to 1.37) and child daycare services (OR, 1.18; 95% CI, 0.96 to 1.44) experienced a higher risk of congenital anomalies.

Among male partners of non-employed pregnant women, the common industrial sections included financial and insurance activities, manufacturing, construction, wholesale/retail trade and repair services, real estate, renting and business activities, public administration and defense/compulsory social security, and public/social/personal services (Table 3). The incidence of congenital anomalies was highest when the male partner was employed in manufacturing (6.2%) and in real estate, renting, and business activities (6.2%), and lowest when employed in financial intermediation (5.8%). Among the seven common industrial sections for male partners, those employed in manufacturing (OR, 1.07; 95% CI, 0.93 to 1.22), real estate, renting, and business activities (OR, 1.10; 95% CI, 0.95 to 1.27), and public administration and defense/compulsory social security (OR, 1.08; 95% CI, 0.93 to 1.25) exhibited risks similar to financial intermediation. In the subgroup analysis of six common industrial divisions, risk estimates were generally imprecise across industries.

## DISCUSSION

This study observed that maternal employment during pregnancy is associated with a higher risk of non-chromosomal congenital anomalies after adjusting for individual risk factors. Specifically, employment in general hospitals and child daycare services was linked with a higher risk of congenital anomalies compared to low-risk industrial divisions. This elevated risk was not observed among male partners employed in semiconductor-manufacturing machinery production or software development and supply. Our findings provide evidence of a positive association between maternal employment in certain industries and the occurrence of congenital anomalies.

Our results are generally consistent with previous studies [10]. A meta-analysis revealed that healthcare workers were more likely to experience certain congenital abnormalities, particularly affecting the neurological and musculoskeletal systems [11]. One key consideration is the potential role of chemical and biological exposures in these occupational settings. For instance, women exposed to anesthetic gases have shown increased risks of fetal abnormalities [12]. Healthcare workers are also at higher risk of intrauterine infection due to exposure to potentially infectious blood, tissues, secretions, and other body fluids [13]. Consequently, women employed in general hospitals may face greater risks because of exposure to chemical and biological teratogens [14,15].

Psychosocial and ergonomic stressors are also important factors. Healthcare workers and educators often experience high levels of job-related stress, irregular work hours, and prolonged standing, all of which have been associated with adverse pregnancy outcomes [16,17]. These factors may independently contribute to fetal developmental risks apart from direct chemical or biological exposures. Our study did not assess specific chemical exposures, highlighting the need for further research using biological or environmental monitoring.

Our relative risk estimates for congenital anomalies did not differ significantly across the common industries employing male workers. Previous research has linked paternal employment in semiconductor manufacturing and electronics with a higher risk of congenital anomalies in offspring [18]. However, in our study, the risk estimates for paternal employment across industrial classifications were generally imprecise compared to those for the financial industry. One study in Taiwan reported higher risks of deaths with congenital anomalies and heart anomalies among offspring of male semiconductor workers [19]. Another study found that male electronics workers exposed to organic solvents had a higher risk of congenital abnormalities, particularly heart defects [20]. We attribute the comparable risk observed between semiconductor manufacturing workers and financial industry employees to our use of industrial classification for assessing occupational exposure, a method that may be less precise than alternatives such as surveys. Some male workers in semiconductor manufacturing may have held white-collar positions, potentially offsetting any positive association between the industry and congenital anomalies. Given these inconsistencies, the potential increased risk of fetal anomalies associated with paternal employment in high-risk industries warrants further investigation.

This study has several limitations. First, our analysis did not consider genetic abnormalities. Chromosomal anomalies were examined by industrial classification as a negative control analysis, which may support our assumptions. However, the number of cases classified as chromosomal anomalies was extremely limited, likely because selective termination often occurs following prenatal screening that detects such anomalies [21]. Similarly, structural anomalies might have been underestimated because our linked dataset only included live births; pregnancies with severe structural anomalies may have ended in termination or in utero fetal death. Second, we identified offspring diagnoses made within 1-3 years of birth, potentially excluding congenital anomalies detected later. However, because most major structural congenital anomalies are identified early in life, we believe any bias from the short observation period would be minimal. Third, residual confounding by unmeasured personal or environmental factors cannot be ruled out. Lastly, our study population may not be generalizable to other geographical or regulatory contexts.

This study demonstrates that maternal employment during pregnancy—particularly in general hospitals—is associated with an increased risk of non-chromosomal congenital anomalies in offspring. These findings suggest a need for targeted interventions and workplace safety measures to mitigate risks associated with specific occupational exposures. Further research is required to elucidate the mechanisms underlying these associations and to inform policies aimed at protecting prenatal health in both maternal and paternal occupational settings.

## Figures and Tables

**Figure f1-epih-47-e2025018:**
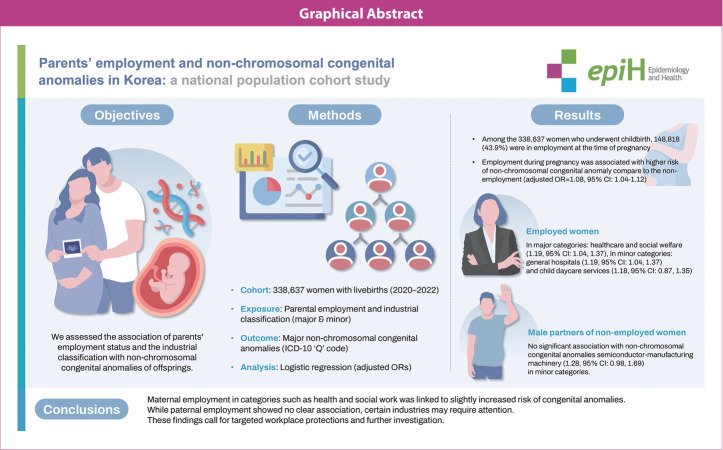


**Table 1.** Demographic and clinical characteristics of Korean women with a livebirth in 2020-2022

Characteristics	Employed (n=148,818)	Not employed	
		Not employed (n=189,819)	Pregnant women with employed male partners (n=108,545)^1^
Maternal age (yr)			
20-24	3,761 (2.5)	11,412 (6.0)	5,783 (5.3)
25-29	37,927 (25.5)	41,573 (21.9)	23,186 (21.4)
30-34	69,199 (46.5)	76,930 (40.5)	45,640 (42.0)
35-39	33,173 (22.3)	51,193 (27.0)	29,508 (27.2)
≥40	4,758 (3.2)	8,711 (4.6)	4,428 (4.1)
Residence			
Seoul capital area^2^	82,483 (55.4)	94,858 (50.0)	54,346 (50.1)
History of smoking	3,061 (2.1)	3,188 (1.7)	1,219 (1.1)
Pre-pregnancy obesity	17,631 (11.8)	11,826 (6.2)	7,194 (6.6)
Income level (%)			
Most deprived (0-30)	42,252 (28.4)	36,304 (19.1)	16,464 (15.2)
Moderately deprived (30-60)	50,213 (33.7)	40,861 (21.5)	18,950 (17.5)
Less deprived (60-80)	40,570 (27.3)	63,887 (33.7)	41,055 (37.8)
Least deprived (80-100)	15,781 (10.6)	48,767 (25.7)	32,076 (29.6)
Presence of maternal disability	49 (0.0)	949 (0.5)	402 (0.4)
Presence of maternal obstetric comorbidity	57,227 (38.5)	84,133 (44.3)	49,844 (45.9)

Values are presented as number (%).

1 The National Health Information Service data provide the employment and industrial classification data of the subscribers only; Data of employment and industrial classifications of male partners were available only when the mothers are not employed; The 108,545 pregnant women with employed male partners are a subset of the 189,819 women who were not employed.

2 Seoul capital area includes Seoul, Incheon, and Gyeonggi Province where the more than 48% of national population dwell.

**Table 2.** Incidence and ORs of congenital anomalies among employed women by employment and common industrial classifications

Employment status/common industrial classification among employed women^1^	No. of children with congenital anomalies (incidence %)^2^	Crude OR (95% CI)	Adjusted OR (95% CI)
Employment at the time of pregnancy			
Employed (n=148,818)	8,857 (6.0)	1.00 (0.97, 1.03)	1.08 (1.04, 1.12)
Non-employed (n=108,545)	11,281 (10.4)	1.00 (reference)	1.00 (reference)
Common industrial sections (major categories)			
Financial and insurance activities (n=17,052)	993 (5.8)	1.00 (reference)	1.00 (reference)
Health/social work (n=29,399)	1,878 (6.4)	1.06 (0.97, 1.17)	1.11 (1.06, 1.22)
Manufacturing (n=21,426)	1,254 (5.9)	1.00 (0.89, 1.13)	1.03 (0.87, 1.22)
Education (n=20,158)	1,245 (6.2)	1.00 (0.91, 1.10)	1.03 (0.93, 1.13)
Wholesale/retail trade and repair services (n=18,979)	1,091 (5.7)	0.98 (0.87, 1.11)	1.00 (0.91, 1.11)
Real estate, renting and business activities (n=17,052)	993 (5.8)	0.94 (0.85, 1.04)	1.01 (0.91, 1.12)
Public/social/personal service (n=9,844)	572 (5.8)	1.02 (0.91, 1.14)	1.07 (0.89, 1.29)
Common industrial divisions (minor categories)			
Financial intermediation (n=2,924)	179 (6.1)	1.00 (reference)	1.00 (reference)
General hospitals (n=9,697)	682 (7.0)	1.15 (1.00, 1.07)	1.19 (1.04, 1.37)
Pre-primary education (n=7,491)	460 (6.1)	0.96 (0.83, 1.10)	1.03 (0.88, 1.21)
General public service (n=5,367)	314 (5.9)	1.03 (0.89, 1.19)	1.01 (0.86, 1.18)
Manufacture of office machinery and equipment (n=2,590)	155 (6.0)	0.96 (0.80, 1.14)	1.01 (0.84, 1.21)
Primary schools (n=2,717)	176 (6.5)	1.00 (0.91, 1.14)	1.02 (0.85, 1.22)
Child daycare services (n=1,759)	123 (7.0)	1.12 (0.92, 1.35)	1.18 (0.96, 1.44)

OR, odds ratio; CI, confidence interval.

1 Occupational groups with >5% of total pregnant women were selected for analysis; The total number of these groups comprised 90% of all women’s occupations.

2 Major congenital anomalies only.

**Table 3.** Incidence and ORs of congenital anomalies among male partners of non-employed women by employment and common industrial classifications

Employment status/common industrial classification among male partners of non-employed women^1^	No. of children with congenital anomalies (incidence %)^2^	Crude OR (95% CI)	Adjusted OR (95% CI)
Common industrial sections (major categories)			
Financial and insurance activities (n=11,643)	685 (5.8)	1.00 (reference)	1.00 (reference)
Manufacturing (n=36,933)	2,292 (6.2)	1.07 (0.34,1.23)	1.07 (0.93, 1.22)
Construction (n=7,713)	480 (6.2)	1.00 (0.86, 1.17)	1.02 (0.88, 1.19)
Wholesale/retail trade and repair services (n=14,294)	837 (5.9)	0.96 (0.84, 1.11)	1.01 (0.86, 1.24)
Real estate, renting & business activities (n=11,643)	2,618 (6.2)	1.06 (0.91, 1.22)	1.10 (0.95, 1.27)
Public administration and defense; compulsory social security (n=9,080)	551 (6.1)	1.04 (0.90, 1.20)	1.08 (0.93, 1.25)
Public/social/personal service (n=6,494)	376 (5.8)	0.97 (0.83, 1.13)	1.00 (0.85, 1.17)
Common industrial divisions (minor categories)			
Financial intermediation (n=1,454)	96 (6.2)	1.00 (reference)	1.00 (reference)
Manufacture of office machinery and equipment (n=4,753)	308 (6.5)	1.09 (0.90, 1.32)	1.10 (0.89,1.31)
Manufacture of semiconductor-manufacturing machinery (n=801)	65 (8.1)	1.29 (0.98, 1.69)	1.28 (0.98, 1.69)
Electrical works (n=656)	51 (7.8)	1.10 (0.82, 1.50)	1.13 (0.83, 1.54)
General public service (n=2,521)	164 (6.5)	1.10 (0.89, 1.36)	1.08 (0.88, 1.34)
Defense activities (n=3,990)	231 (5.8)	0.96 (0.78, 1.17)	1.10 (0.84, 1.44)
Software development and supply (n=1,694)	111 (6.6)	1.16 (0.93, 1.46)	1.19 (0.94, 1.50)

OR, odds ratio; CI, confidence interval.

1 Occupational groups with >5% of total male partners of pregnant women were selected for analysis; The total number of these groups comprised 90% of all male partner’s occupations.

2 Major congenital anomalies only.

## Supplementary materials

Supplementary Material 1. Flow of selecting study population from the employed National Health Information Service (NHIS) subscribers and dependents of the employed NHIS subscribers.
