# Supplementary material for: Parents’ employment and non-chromosomal congenital anomalies in Korea: a national population cohort study
Source: Epidemiol Health. 2025 Apr 10;47:e2025018. doi: 10.4178/epih.e2025018 (PMC12425695; doi:10.4178/epih.e2025018)

Supplementary Material 1. Flow of selecting study population from the employed National Health Information Service (NHIS) subscribers and dependents of the employed NHIS subscribers.


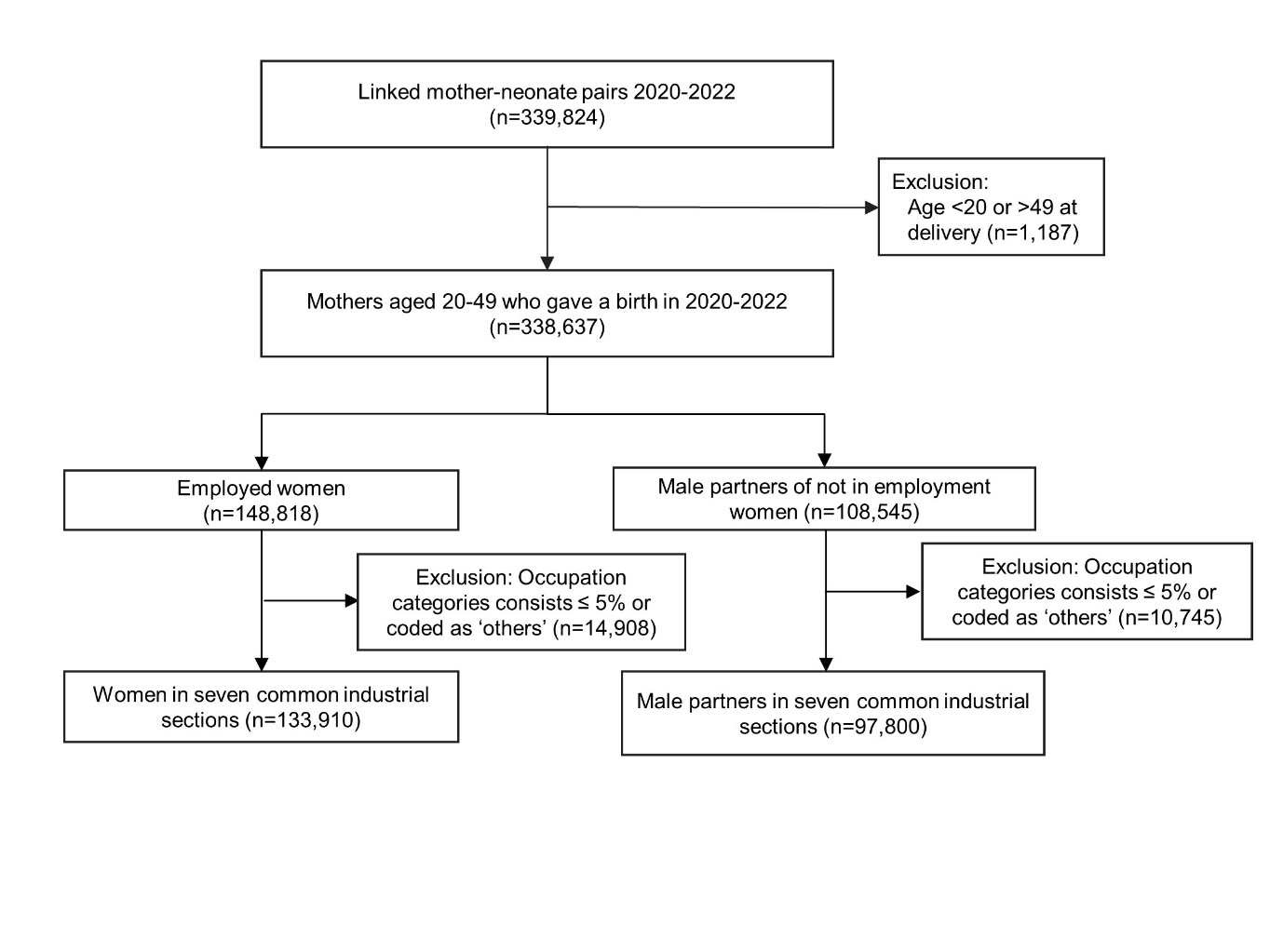

Supplement: Supplementary Material 1. — Flow of selecting study population from the employed National Health Information Service (NHIS) subscribers and dependents of the employed NHIS subscribers. [file epih-47-e2025018-Supplementary-1.docx]
